# Neonatal Abusive Head Trauma without External Injuries: Suspicion Improves Diagnosis

**DOI:** 10.3390/children9060808

**Published:** 2022-05-30

**Authors:** Seokwon Yoon, Juyoung Lee, Yong Hoon Jun, Ga Won Jeon

**Affiliations:** 1 Department of Pediatrics, Inha University Hospital, Incheon 22332, Korea; yoonjoon3@gmail.com (S.Y.); juyounglee@inha.ac.kr (J.L.); neojun@inha.ac.kr (Y.H.J.); 2 Department of Pediatrics, Inha University College of Medicine, Incheon 22332, Korea

**Keywords:** shaken baby syndrome, diffuse axonal injury, child abuse, subdural hemorrhage, brain injuries, brain edema, abusive head trauma

## Abstract

The term “shaken baby syndrome” has been replaced by “abusive head trauma (AHT)” based on the mechanism of injury. The reported mortality rate of AHT ranges from 10% to 30%. Up to two-thirds of survivors suffer from serious long-term disabilities. Thus, an expeditious and accurate diagnosis is crucial to prevent further abuse that might result in death or serious disabilities. It remains a challenge for physicians to diagnose AHT when parents do not give a history of trauma in preverbal infants without any external signs. Here, we report a case of a 14-day-old boy who presented with a febrile convulsion without evident external injuries nor history of trauma according to his parents. He was diagnosed with AHT based on MRI findings of subacute subdural hemorrhage, multiple cortical hemorrhages, cerebral edema, and diffuse axonal injury. In conclusion, health care providers should keep in mind that the history of trauma provided by the parents or caregivers might not always be true and that reasonable suspicion of abuse is the most important in the diagnosis of AHT, although neuroimaging plays a pivotal role. Reasonable suspicion of AHT in combination with a thorough physical examination, neuroimaging, and skilled neuroradiologist can improve diagnosis and help victims in a timely manner.

## 1. Introduction

First introduced about 40 years ago [1], the term “shaken baby syndrome” has been used for decades. Regarding the mechanism of shaken baby syndrome, it has been revealed that not only violent shaking, but also blunt impact or a combination of shaking and blunt impact can cause brain injury to the intracranial contents or skull. Thus, the term abusive head trauma (AHT) has been adopted as a more precise meaning based on the recommendation of the American Academy of Pediatrics [2]. The reported mortality rate of babies with AHT ranges from 10% to 30%. Two-thirds of survivors suffer from serious long-term disabilities [3]. Those disabilities include seizures, cerebral palsy, cortical blindness, and behavior, intellectual, and learning problems resulting from a secondary brain injury by hypoxia, ischemia, or inflammation [4,5]. Thus, an expeditious and accurate diagnosis is crucial to prevent further abuse that might result in death or serious disabilities.

Important diagnostic tools for AHT include the presence of the history of trauma, abnormal signs upon physical examination, and neuroimages that commonly suggest subdural hemorrhage (SDH), retinal hemorrhage, and multiple bone fractures [6,7]. However, there are no established diagnostic criteria for AHT. Since parental history of trauma is one of the most important components in establishing the diagnosis of AHT, we might miss the diagnosis of AHT especially when parents do not give a history of trauma in a baby with a lack of evident external injuries [7,8]. Clinical findings can be quite heterogeneous. Up to 40% of infants diagnosed with AHT do not have any external injuries [9,10]. Health care providers must be aware of the possibility of AHT. Reasonable suspicion of abuse is required.

Here, we report the diagnosis of neonatal AHT for a baby without external injuries or skull fractures and suggest actions to be taken.

## 2. Case Presentation

A 14-day-old boy was admitted to the neonatal intensive care unit through the emergency room with a suspicious febrile convulsion. According to his parents’ statement, two days before admission, he had seizure-like symptoms including twitching of the mouth, roving eye movements, and eyelid blinking. The parents brought him to a local pediatrician’s clinic. He was expected to be followed up with because of the uncertainty of neonatal seizure. On the day of admission, he had a fever of 38.2 °C with seizures similar to the previous one. Thus, the parents brought him to the emergency room of our hospital.

He was born at 40^+5^ weeks of gestation with a body weight of 3270 g (44 percentile, z-score: −0.16) via vaginal delivery. There were no prenatal or postnatal problems. He was the first baby of a healthy two-parent Korean family. At admission, his body weight was 3420 g (150 g of weight gain for postnatal two weeks, 17 percentile, z-score: −0.94) with a height of 51 cm (25 percentile, z-score: −0.68) and a head circumference of 36.5 cm (70 percentile, z-score: 0.52). Vital signs at admission were as follows: heart rate, 144 beats/min; blood pressure, 67/42 mmHg; respiratory rate, 48 breaths/min; and body temperature, 38.5 °C. He did not have irritability, lethargy, or vomiting. His mental status was alert. His pupil size and light reflex were normal. He did not have any external injuries suggesting abuse. The parents denied a history of trauma.

Laboratory tests of serum at admission were within normal limits as follows: hemoglobin, 12.4 g/dL; white blood cells (WBC), 19,980/µL (neutrophil 62%); platelets, 582,000/µL; C-reactive protein, 0.21 mg/dL; procalcitonin, 0.20 ng/mL; sodium/potassium/chloride, 136.7/5.04/100.1 mEq/L; aspartate aminotransferase/alanine transaminase, 33/13 IU/L; and amylase/lipase, 10/8 U/L. He had normal coagulation profiles. His urinalysis at admission was positive for leukocyte esterase, showing WBC ≥ 16~20/high power field with microscopy. His cerebrospinal fluid (CSF) was bloody by lumbar puncture at admission, showing 1,260,000/mm^3^ of red blood cells and 2350/mm^3^ of WBCs. With the impression of a urinary tract infection, empirical antibiotics were prescribed. The fever subsided. No more seizure-like movements were detected during hospitalization. 

Electroencephalography revealed a normal stage one sleep record. Brain ultrasonography on the fourth day of admission revealed increased echogenicity in the right caudothalamic groove suggesting germinal matrix hemorrhage, increased echogenicity through the cortex in bilateral hemispheres, and brain swelling suggesting meningoencephalitis. For further evaluation, we performed brain magnetic resonance imaging (MRI) on the fifth day of admission. His brain MRI showed subacute SDH along the posterior aspect of the falx cerebri and occipital convexity, subarachnoid hemorrhage in the sylvian fissure and along the sulci of both cerebral vertices, multiple cortical hemorrhages with edema, microhemorrhages in the left frontal lobe, and diffuse axonal injury in the periventricular white matter, corpus callosum, right thalamus, and both internal and external capsules.

**Figure 1:**
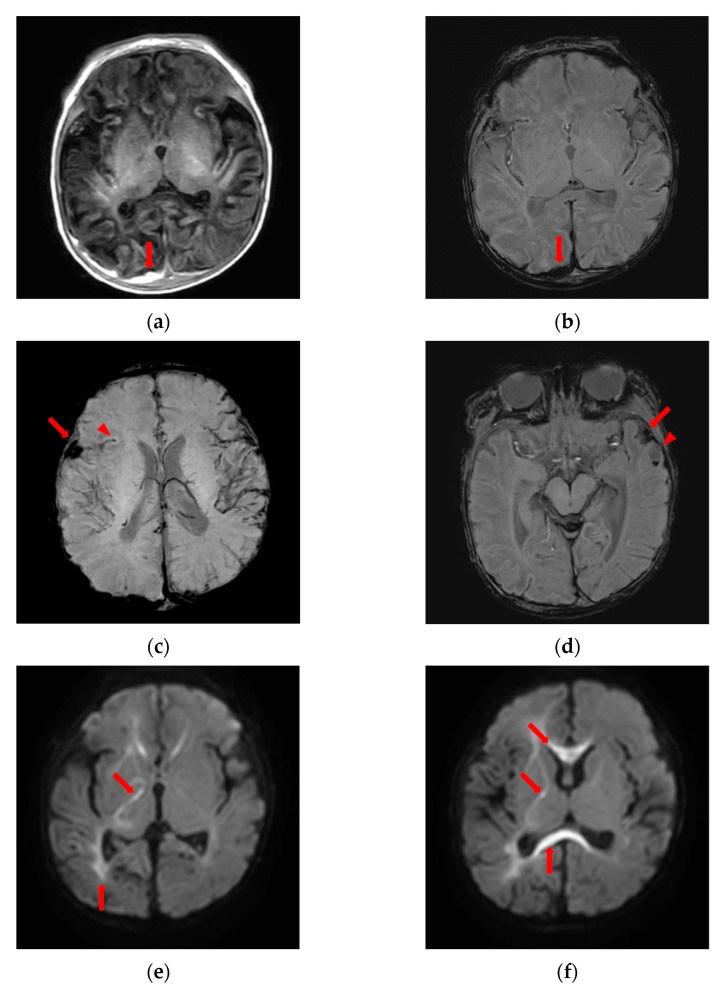
Brain magnetic resonance imaging suggesting abusive head trauma. (**a**) High signal intensity on T1-weighted image and (**b**) low signal intensity on susceptibility-weighted image, subdural fluid collection along the posterior aspect of the falx cerebri and occipital convexity implying subacute subdural hemorrhage (arrows); (**c**) low signal intensity in the right sylvian cistern (arrow), right sylvian fissure (arrowhead) and (**d**) left sylvian cistern (arrow) implying subarachnoid hemorrhage on susceptibility-weighted image; (**d**) low signal intensity with fluid-fluid level (arrowhead) along the anterior side of left temporal lobe implying acute hemorrhage on susceptibility-weighted image; (**e**,**f**) diffusion restriction in periventricular white matter, corpus callosum, right thalamus, and internal and external capsules on diffusion-weighted image (arrows).

We informed the parents of these findings from the brain MRI and asked them again about the history of trauma. However, they denied trauma emphatically. With the possibility of AHT, we performed additional evaluations to detect abusive injuries. Radiographs of the skull, ribs, upper extremities, and lower extremities showed no abnormal findings such as fractures. Fundoscopy revealed no abnormal findings such as retinal hemorrhage. Abdominal ultrasonography revealed no abnormal findings in the liver, spleen, adrenal gland, pancreas, or intestine. As AHT was strongly implied by a skilled neuroradiologist based on his brain MRI findings, namely, the subacute SDH, multiple cortical hemorrhages, cerebral edema, and diffuse axonal injury, we immediately reported this case to the National Investigative Agency, which investigated the parents for child abuse. The parents were accused and charged with child abuse. The baby was separated from the parents and admitted to a child welfare facility (see Figure 1). 

During follow-up, his weight increased within normal ranges until 2 months of age. However, his head growth was poor: 36.5 cm (70 percentile, z-score: 0.52) at postnatal 14 days, 36.6 cm (17 percentile, z-score: −0.94) at postnatal 37 days, and 37.0 cm (7 percentile, z-score: −1.45) at postnatal 53 days. Brain computed tomography taken at 2 months of age revealed encephalomalacia in the right parieto-occipital cerebral hemisphere with foci of hemorrhage or calcifications, widened CSF spaces (right > left), and ventriculomegaly suggesting brain atrophy. This baby is being followed up regularly for timely and proper intervention. Although he did not present epilepsy until 2 months of age at follow-up, he is at great risk for long-term neurodevelopmental impairments such as mental, motor, or social developmental delay including cerebral palsy, blindness, and behavior, intellectual, or learning problems resulting from brain injury. Therefore, he needs to be followed up to assess long-term neurodevelopmental outcomes.

## 3. Discussion

A worldwide increase of child abuse has been an emerging burden and challenge for child health systems. A total of 30,905 cases of child abuse were reported in Korea in 2020, showing an extreme increase by 164% compared to 11,715 cases in 2015. Of them, 43 infants died in Korea in 2020 due to abuse. Since 82.7% of perpetrators were victims’ own parents, it is not easy to diagnose or interrupt the abuse [11]. Further, a total of 10% of perpetrators were other caregivers such as babysitters, caregivers in infant care facilities, or cohabitants of the mother or father, and a total of 5% of them were victims’ own relatives [12]. Child abuse occurred most frequently among two-parent families, single-parent families, and remarried families [12]. Further, child abuse was more common among boys, young infants (younger than one year), or in low-income families than among girls, toddlers, or in high-income families, which was associated with infants crying and the subsequent caregiver’s rage against them [13]. The majority of AHT cases have been found in children younger than two years, especially those who are younger than six months whose brains are the most susceptible [14]. Thus, AHT is more fatal than any other abuses. It can leave the child severely disabled for life [15]. Vomiting, irritability, lethargy, and fever are common symptoms of a variety of conditions seen in young infants, including AHT. Therefore, it is difficult to diagnose AHT in young infants with these nonspecific symptoms. Furthermore, when parents or caregivers do not give a history of trauma in preverbal infants without showing external signs, it remains a challenge for physicians to diagnose AHT with confidence. Thus, AHT can be misdiagnosed to a less-serious condition [8]. Delayed proper management and separation from the perpetrators due to misdiagnosis of abuse might lead to more serious conditions or death [8]. For this reason, health care providers should strongly suspect AHT if the baby manifests unexplained seizures, irritability, change of mental status, apnea, breathing difficulties, vomiting without fever, or diarrhea [16]. 

Neuroimaging plays a crucial role to diagnose AHT. Interpretation of neuroimaging with skilled neuroradiologists, not non-neuroradiologists, can improve the detection rate of AHT [17]. If there is an SDH on the neuroimaging of infants, AHT should be suspected [18,19]. SDH, multiple interhemispheric, convexity hemorrhages, and cerebral edema on neuroimaging are strongly associated with AHT, not non-abusive head trauma [20]. In addition, a fundoscopic examination to detect retinal hemorrhage and skeletal survey to detect fractures should be performed [21]. Coagulation tests and an evaluation of injuries in the liver, spleen, adrenal gland, pancreas, and intestine should be performed to detect abdominal trauma [6,22]. Differential diagnosis of AHT includes accidental head injury (non-abusive head trauma), infection, venous sinus thrombosis, coagulopathies, metabolic diseases, arachnoid cyst, osteogenesis imperfecta, benign enlargement of the subarachnoid spaces, and so on [23]. The diagnosis of AHT can be established by excluding other possible diagnoses.

Our patient presented with a suspicious febrile convulsion without evident external injuries. AHT was suspected based on findings from the brain MRI. However, the parents denied trauma. There were no other clues for abusive injuries, such as retinal hemorrhage, bone fractures, bruises, or abdominal trauma. Thus, it was a challenge for us to diagnose AHT with confidence. However, his brain MRI findings, namely, subacute SDH, multiple cortical hemorrhages, cerebral edema, and diffuse axonal injury, were identified by a skilled neuroradiologist and strongly implied AHT. In Korea, with a recent revision of the law, if there is suspected child abuse, health care providers are obliged to report it to the investigative agency. Thus, we immediately reported this case to the National Investigative Agency. Finally, the parents were found to be guilty and charged with child abuse. The baby was separated from the parents without further abuse. If we hesitated to report this case to the investigative agency, we might not have been able to prevent the continuing abuse, which could have led to the loss of this baby. Thus, health care providers should keep in mind that the history of trauma provided by the parents or caregivers might not always be true and that symptoms of abusive injuries, especially in young infants, can be nonspecific.

In conclusion, our patient was an abused baby who presented with a seizure without any external injuries. He was diagnosed with AHT based on the instinctive suspicion of a physician and a skilled neuroradiologist. Although neuroimaging plays a pivotal role in the diagnosis of AHT, reasonable suspicion of abuse is the most important for primary health care providers. Reasonable suspicion of AHT in combination with a thorough physical examination, neuroimaging, and skilled neuroradiologist can improve diagnosis and help victims in a timely manner.

## Data Availability

All data and material analyzed in this study are included in this published article.
